# Electron-induced ligand loss from iron tetracarbonyl methyl acrylate

**DOI:** 10.3762/bjnano.15.66

**Published:** 2024-07-03

**Authors:** Hlib Lyshchuk, Atul Chaudhary, Thomas F M Luxford, Miloš Ranković, Jaroslav Kočišek, Juraj Fedor, Lisa McElwee-White, Pamir Nag

**Affiliations:** 1 J. Heyrovský Institute of Physical Chemistry, Czech Academy of Sciences, Dolejškova 3, 182 23 Prague, Czech Republic; 2 Department of Physical Chemistry, University of Chemistry and Technology, Technická 5, 16628 Prague, Czech Republic; 3 Department of Chemistry, University of Florida, Gainesville, Florida 32611-7200, United States

**Keywords:** electron collision, focused electron beam-induced deposition (FEBID), FEBID precursor, iron tetracarbonyl methyl acrylate

## Abstract

We probe the separation of ligands from iron tetracarbonyl methyl acrylate (Fe(CO)_4_(C_4_H_6_O_2_) or Fe(CO)_4_MA) induced by the interaction with free electrons. The motivation comes from the possible use of this molecule as a nanofabrication precursor and from the corresponding need to understand its elementary reactions fundamental to the electron-induced deposition. We utilize two complementary electron collision setups and support the interpretation of data by quantum chemical calculations. This way, both the dissociative ionization and dissociative electron attachment fragmentation channels are characterized. Considerable differences in the degree of precursor fragmentation in these two channels are observed. Interesting differences also appear when this precursor is compared to structurally similar iron pentacarbonyl. The present findings shed light on the recent electron-induced chemistry of Fe(CO)_4_MA on a surface under ultrahigh vacuum.

## Introduction

In recent years, a wave of interest in the electron-induced loss of ligands from organometallic and coordination compounds appeared, which has been motivated by the need to understand focused electron beam-induced deposition (FEBID). FEBID is an emerging method for the fabrication of 3D nanostructures. It relies on the local decomposition of precursors in the focal area of an electron beam [1–4]. In the case of deposition of metals, the interaction with the electrons should ideally lead to a cleavage of all metal–ligand bonds and leave a pure metallic deposit. However, this has been achieved only for a handful of metals and their precursors. The actual deposits are often contaminated by a high amount of impurities [5]. Several reasons have been put forward for this incomplete dissociation [6]. One of them is the presence of a plume of slow electrons in the deposition region. While the primary focused beam typically has an energy of tens of kiloelectronvolts, the distribution of secondary backscattered electrons often peaks at tens of electronvolts [7]. Interaction of precursors with these secondary electrons also leads to metal–ligand bond cleavage, which is often incomplete and leads to the accumulation of organic contamination in the final deposit.

**Figure 1:**
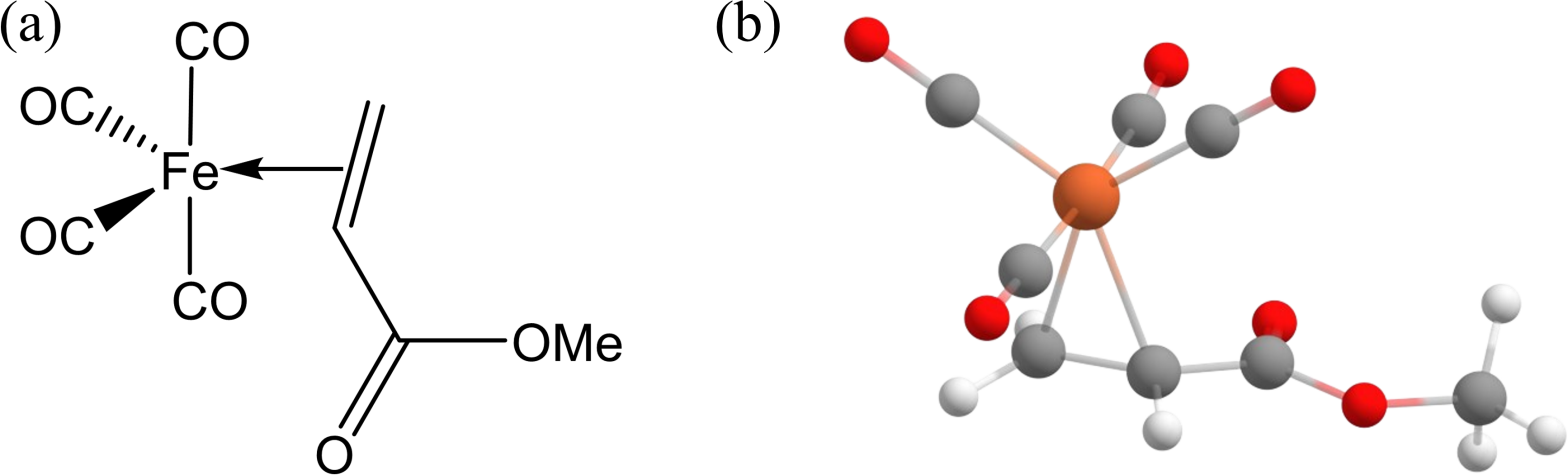
(a) Structure of iron tetracarbonyl methyl acrylate and (b) optimized geometry of iron tetracarbonyl methyl acrylate.

So far, the above interest has focused mainly on the well-established precursors. In this paper, we probe a novel precursor, namely, iron tetracarbonyl methyl acrylate, Fe(CO)_4_(C_4_H_6_O_2_), further denoted as Fe(CO)_4_MA. The structure of Fe(CO)_4_MA is shown in Figure 1. This precursor is related to iron pentacarbonyl, Fe(CO)_5_, with one ligand replaced with an olefinic methyl acrylate ligand. Recently, Fe(CO)_4_MA has been utilized for electron-induced deposition under ultrahigh-vacuum conditions [8]. The deposits had an Fe/C/O composition similar to those obtained from Fe(CO)_5_, which was surprising since the methyl acrylate ligand has a high carbon content. This opens a fundamental question of how much can a change in one ligand change the outcome of electron-induced reactions? This is basically impossible to predict a priori since several effects come into play, for example, change in bond dissociation energies, electron density at the metal, and dipole moment. Of the possible experimental approaches to address this question, a crossed-beam gas-phase experiment represents perhaps the “cleanest” approach since it probes the reaction of one precursor molecule with at most one electron, without environmental influences (e.g., precursor–precursor or precursor–substrate effects).

The possibility of making iron nanostructures is important mainly because of their magnetic properties, which could be used in nanosensing applications [9]. Common FEBID precursors for iron are Fe(CO)_5_, Fe_2_(CO)_9_, Fe_3_(CO)_12_, and Fe(C_5_H_5_)_2_. The first one, iron pentacarbonyl, has attracted perhaps the highest amount of attention from the point of view of the elementary reaction with electrons. The probable reasons are its availability (it is a common precursor for iron-containing compounds in organometallic synthesis), ease of handling (it is a volatile liquid at standard conditions), and relatively simple structure, which allows for advanced theoretical description. The ligand loss from Fe(CO)_5_ in the gas phase has been probed with respect to its dissociative ionization [10] and dissociative electron attachment [11–12]; there is even information available on its electronic excitation, which is the first step towards neutral dissociation [11]. The gas-phase studies have been complemented by surface-based investigations, where the electron-induced ligand loss has been probed by XPS [13], ion desorption [14], IR spectroscopy [15], or cluster-beam studies [16–18]. The ligand loss has also been probed by ion impact, both in the gas phase [19] and on the surface [13], and, theoretically, by advanced reactive force field molecular dynamics simulations [20].

Here we focus on two electron-induced dissociative channels of Fe(CO)_4_MA, namely, dissociative ionization and dissociative electron attachment (DEA). We focus on the electron energy range below 20 eV. Data from two complementary experimental setups are combined with quantum chemical calculations to provide information about ligand loss pathways. The results are brought into context with the deposition experiments of Boeckers and coworkers [8].

## Methods

### Synthesis of Fe(CO)_4_(C_4_H_6_O_2_)

#### General considerations

All reactions were carried out in an inert atmosphere of dinitrogen using either Schlenk or glovebox techniques. Glassware was flame-dried or oven-dried for at least 3 h before use. Solvents (i.e., benzene and hexane) were purified using an MBraun MB-SP solvent purification system and stored over 3 Å molecular sieves for 24 h before use. Diiron nonacarbonyl was purchased from Fisher Scientific, and methyl acrylate was purchased from MilliporeSigma and used without further purification. Deuterated solvent (chloroform-*d*) for NMR was purchased from Cambridge Isotopes Lab and was stored over 4 Å molecular sieves for 24 h prior to use. ^1^H nuclear magnetic resonance spectra (NMR) were obtained on a Bruker 400 MHz spectrometer. IR spectra were obtained on a PerkinElmer Spectrum ONE FTIR spectrometer using a solution cell equipped with NaCl windows and a path length of 1.0 mm.

#### Synthesis

The compound was synthesized according to the reported literature procedure and characterized by comparison to literature data [21]. In a nitrogen-filled glovebox, a 100 mL Schlenk flask equipped with a stir bar was charged with Fe_2_(CO)_9_ (0.50 g, 1.37 mmol) in 20 mL of benzene. The Schlenk flask was taken out of the glovebox and connected to the Schlenk line, followed by the addition of methyl acrylate (0.12 mL, 1.37 mmol) under an inert atmosphere of dinitrogen. The reaction mixture was stirred at 45 °C for 4 h, and the color of the solution gradually changed from yellow to brown. After 4 h, the solvent was removed under vacuum, and the resulting crude solid product was sublimed at room temperature at 700 mTorr to yield the crystalline yellow product. Yield: 140 mg, 40%. Purity of the product was assessed using ^1^H NMR and IR spectroscopy. IR (hexane) ν_CO_ (cm^−1^): 2100, 2034, 2020, 1997. ^1^H NMR (400 MHz, CDCl_3_) δ 3.71 (s, 3H), 3.25 (dd, *J* = 11.6, 7.8 Hz, 1H), 2.91 (dd, *J* = 11.6, 2.3 Hz, 1H), 2.62 (dd, *J* = 7.7, 2.3 Hz, 1H).

### Electron collision experiments

Two different experimental setups were used to perform low-energy electron beam-induced dissociation experiments. They are complementary; one of them, the CLUster Beam setup (CLUB), has a much higher mass resolution, while the second one, the trochoidal electron monochromator quadrupole mass spectrometer (TEM-QMS), has a higher energy resolution of the incident electron beam.

#### The CLUB setup

The CLUB experimental setup has been described in detail in previous papers [22–23] and recently used for similar studies with different molecules [24]; hence, only a short overview will be given here. While the apparatus is typically used for molecular beam studies, the setup also allows for the study of isolated gas-phase molecules by introducing them as a background gas into the time of flight (TOF) chamber [25–26].

The sample was kept in a glass container at room temperature (around 25 °C); its vapor was introduced into the interaction chamber via a leak valve and exposed to low-energy electrons produced from a simple magnetically collimated electron gun with a heated tungsten filament. The energy of electrons can be varied between 0 and 70 eV. The base pressure in the chamber is 1 × 10 ^−8^ mbar, and during the measurements the background sample pressure reaches around 4 × 10^−7^ mbar. Product ions were extracted from the interaction region into a reflectron time-of-flight mass spectrometer (RTOF). The RTOF is bipolar and is, thus, able to analyze and detect either cations or anions. The mass resolution of the spectrometer is *M*/Δ*M* = 4000. The electron gun is not monochromated and has an incident electron beam resolution of around 1 eV [27]. It should be noted that the electron beam is difficult to control at low energies below 2 eV. The abundance of electrons below this energy is uncertain since a large fraction of them does not leave the collision region to the Faraday cup, and the energy resolution is also deteriorated. The absolute energy scale of the electron beam was calibrated by measuring and fitting the 4.4 and 8.2 eV DEA resonance peaks of O^−^ anions produced from CO_2_ molecules.

#### TEM-QMS setup

The TEM-QMS setup was originally constructed and operated at the University of Fribourg, Switzerland [28], and was later moved to Prague and modified [29]. A continuous electron beam is produced from a directly heated yttrium-coated iridium cathode, and a trochoidal electron monochromator is used to narrow down the electron energy distribution and to produce a quasi-monochromatic electron beam. A gas-phase effusive beam was produced by introducing the liquid Fe(CO)_4_MA sample, kept at around 25 °C, in a glass container through the gas inlet system. Electrostatic ion optics lenses extract and guide all produced anions from the interaction region towards a quadrupole mass filter that only lets pass anions with a given mass-to-charge ratio (*m*/*z*). Selected anions are then detected with a channeltron and corresponding counting electronics. Each ion yield curve was obtained individually by scanning the incident electron energy in small steps and measuring the resulting count rate for ions with a given *m*/*z* value.

The incident electron beam energy scale was calibrated using the 4.4 eV resonance peak of O^−^ anions produced via DEA to CO_2_. The electron beam energy resolution was estimated by fitting and extracting the width of the 4.4 eV resonance peak of O^−^/CO_2_; during the present measurement it was around 100 meV. In the given mass range, the quadrupole mass resolution was set to around 100 (*M*/Δ*M*).

### DFT calculations

DFT-based structure optimization calculations have been performed using Gaussian 16 software [30]. All calculations were conducted using the commonly employed hybrid functional B3LYP [31] with a 6-31++G(d,p) [32–33] basis set and included the GD3 empirical dispersion correction [34]. We have optimized the structures of the reactant (neutral Fe(CO)_4_MA) and products (fragment ions and potential neutral co-fragments) generated in the dissociative processes. All fragments with an even number of electrons were assumed to be in singlet spin states, and the fragments with an odd number of electrons were assumed to be in doublet spin states. Threshold energies listed in the tables were obtained as differences of sums of the electronic and zero-point energies of products and reactants as


[1] $$E_{\text{th}}=\sum_{i}^{\text{products}}E_{i}-E\left[\text{Fe}{\left(\text{CO}\right)}_{4}\text{MA}\right].$$

Here, *E_i_* is the sum of electronic and zero point energies of a given fragment, and the sum goes over all charged and neutral fragments. A negative value of threshold energy, thus, corresponds to an exothermic reaction.

## Results and Discussion

### Dissociative ionization

**Figure 2:**
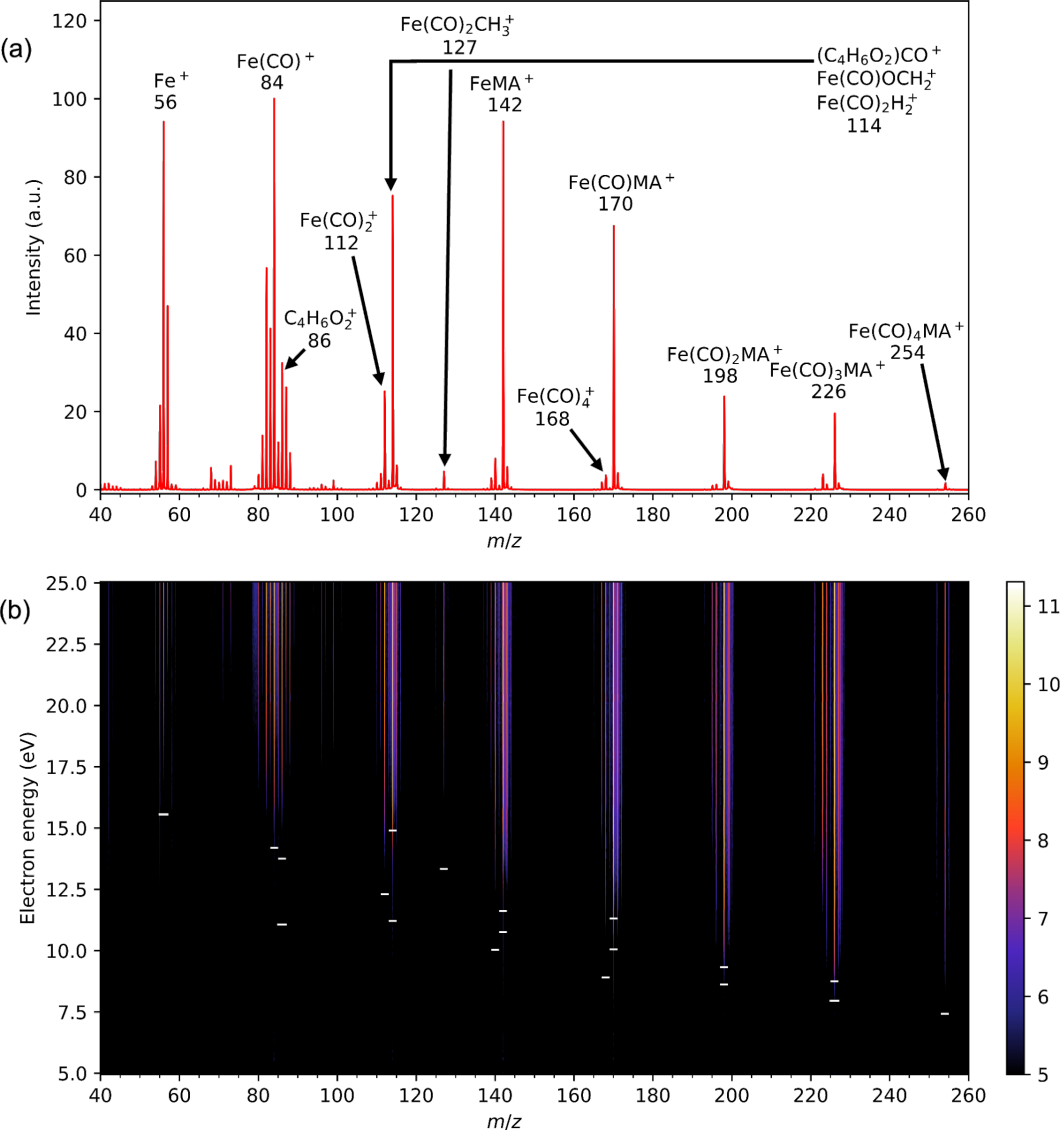
(a) Positive ion mass spectrum of FeCO_4_MA recorded at 70 eV incident electron energy measured using the CLUB experimental setup. (b) 2D plot of the dependence of positive ion mass spectra on the incident electron energy. The logarithm of the ion intensity is color-coded as shown by the color bar on the right. The small horizontal dashes mark the calculated threshold energies from Table 1 below.

Figure 2a shows the positive ion mass spectrum of Fe(CO)_4_MA measured on the CLUB setup at a constant incident electron energy of 70 eV. The mass spectrum shows extensive fragmentation: The parent cation (*m*/*z* = 254) is visible in the spectrum; however, it is very weak. In the high-mass range, there is a strong progression of CO loss channels with one, two, and three carbonyl ligands being removed (*m*/*z* = 226, 198, and 170, respectively). Relative to these, the loss of the methyl acrylate ligand (Fe(CO)_4_^+^, *m*/*z* = 168) has a very low probability. The dominant fragments, however, are the small ones, namely, bare iron ion Fe^+^ and iron with one carbonyl Fe(CO)^+^.

Surprising is the presence of the peak with *m*/*z* = 114, which is relatively strong. This mass may correspond to three different structures, all of them requiring considerable rearrangement during the dissociative ionization process. Formation of Fe(CO)OCH_2_^+^ requires migration of the methoxy group from the methyl acrylate ligand to the iron atom, formation of Fe(CO)_2_H_2_^+^ requires removal of two hydrogen atoms from MA and their bond formation with iron, and, finally, this mass can have a stochiometry of (C_4_H_6_O_2_)CO^+^, two different ligands without the iron atom. We will return to the nature of this fragment below.

There are interesting differences to the 70 eV mass spectrum of iron pentacarbonyl. There, the most abundant fragments are also Fe^+^ and FeCO^+^, and their relative abundance compared to heavier fragments is even stronger that in the case of Fe(CO)_4_MA. When comparing mass spectra from different instruments (e.g., the most recent spectrum of Lacko et al. [10] was recorded with a quadrupole mass filter) one has to keep in mind possible mass-dependent transmissions; however, for iron pentacarbonyl, the strong dominance of Fe^+^ and FeCO^+^ is also visible in all of the older mass spectra (e.g., in the data recommended by NIST [35]). In this sense, the Fe(CO)_4_MA fragment distribution is more uniform, and the relative abundance of heavier fragments is closer to that of Fe^+^ and FeCO^+^. On average, Fe(CO)_4_MA loses fewer ligands per ionization event. We presume that this might be ascribed to a higher number of internal degrees of freedom and, thus, a higher vibrational density of states. It is well established that, upon vertical electron removal, which can create a cation in many different electronic states, the cation relaxes to its electronic ground state on an ultrafast timescale (typically via a series of conical intersections). The excess energy is then transferred into nuclear motion, where it is redistributed among the vibrational degrees of freedom and the ionic complex decays statistically. The methyl acrylate ligand provides a heat bath that absorbs a large amount of excess energy, thus lowering the dissociation degree.

Figure 2b shows the energy dependence of the mass spectra in the threshold electron energy region (5 to 25 eV). The horizontal axis depicts the masses, the vertical axis shows the incident electron energy, and the intensity is color-coded. The intensity profiles along the vertical traces, thus, correspond to the ion yield for a given *m*/*z*. Such a 2D map provides comprehensive information about the energetics and the fragmentation in the threshold region in one picture. At first glance, an intuitively expected effect is visible in the map: The appearance energies of fragment ions increase with the degree of fragmentation since more energy is needed to cleave more bonds (the seeming exception from this rule is the parent ion, *m*/*z* = 254; however, its seemingly high appearance energy is a visual effect caused by a very low signal level on this mass). In Supporting Information File 1, we show the ion yield curves extracted from the 2D map, which clearly show the higher appearance energy with increasing ligand loss.

Table 1 shows the calculated threshold energies of the fragment ions. These energies are marked with short horizontal bars in Figure 2b. For several ionic fragments we have identified two stable structures. One is always derived from the structure of the neutral precursor. In the second one, however, the methyl acrylate ligand has adopted an η^4^ bonding mode, such that the carbonyl moiety interacts with the iron center. A related rearrangement of the ligand has been reported to occur after photochemical CO loss from Fe(CO)_4_MA [36–37]. The cation structures are shown in Supporting Information File 1. Interestingly, in all cases, the second class for fragment ions is energetically more stable (the calculated threshold energies are lower). However, their formation requires a considerable structural rearrangement, and the experimental data (ion yield curves) do not contradict the formation of “standard” structures where all the ligands are bound to the iron atom. However, the ion yield curve for *m*/*z* = 114 clearly sets on well below 14.9 eV, which is the calculated threshold energy for the formation of Fe(CO)OCH_2_^+^. Since the threshold for Fe(CO)_2_H_2_^+^ is even higher (15.22 eV), we conclude that the dominant contribution to this mass comes from the part of the signal originating from the ironless (C_4_H_6_O_2_)CO^+^ complex.

**Table 1:** Calculated threshold energies (in units of eV) of cationic fragments from the reaction Fe(CO)_4_(η^2^-C_4_H_6_O_2_) + e^−^.

*m*/*z*	Ion	Neutral fragments	*E*_th_

254	Fe(CO)_4_(η^2^-C_4_H_6_O_2_)^+^		7.43
226	Fe(CO)_3_(κ^1^-C_4_H_6_O_2_)^+^	CO	7.96, 8.75
198	Fe(CO)_2_(κ^1^-C_4_H_6_O_2_)^+^	2 × CO	8.62^a^, 9.33
170	Fe(CO)(η^2^-C_4_H_6_O_2_)^+^	3 × CO	10.06^a^, 11.31
168	Fe(CO)_4_^+^	(C_4_H_6_O_2_)	8.91
142	Fe(η^4^-C_4_H_6_O_2_)^+^	4 × CO	11.62^a^
142	Fe(CO)_3_H_2_^+^	(C_4_H_4_O_2_), CO	13.01
140	Fe(CO)_3_^+^	(C_4_H_6_O_2_), CO	10.04
127	Fe(CO)_2_CH_3_^+^	(C_3_H_3_O_2_), 2 × CO	13.33
114	(C_4_H_6_O_2_)CO^+^	Fe(CO)_3_	11.21
114	Fe(CO)OCH_2_^+^	3 × CO, C_3_H_4_O	14.90
114	Fe(CO)_2_H_2_^+^	2 × CO, (C_4_H_4_O_2_)	15.22
112	Fe(CO)_2_^+^	2 × CO, (C_4_H_6_O_2_)	12.31
86	C_4_H_6_O_2_^+^	Fe(CO)_4_	11.07
84	Fe(CO)^+^	3 × CO, (C_4_H_6_O_2_)	14.20
56	Fe^+^	4 × CO, (C_4_H_6_O_2_)	15.65

^a^The methyl acrylate ligand is η^4^-bonded such that the carbonyl oxygen interacts with the iron center.

### Dissociative electron attachment

**Figure 3:**
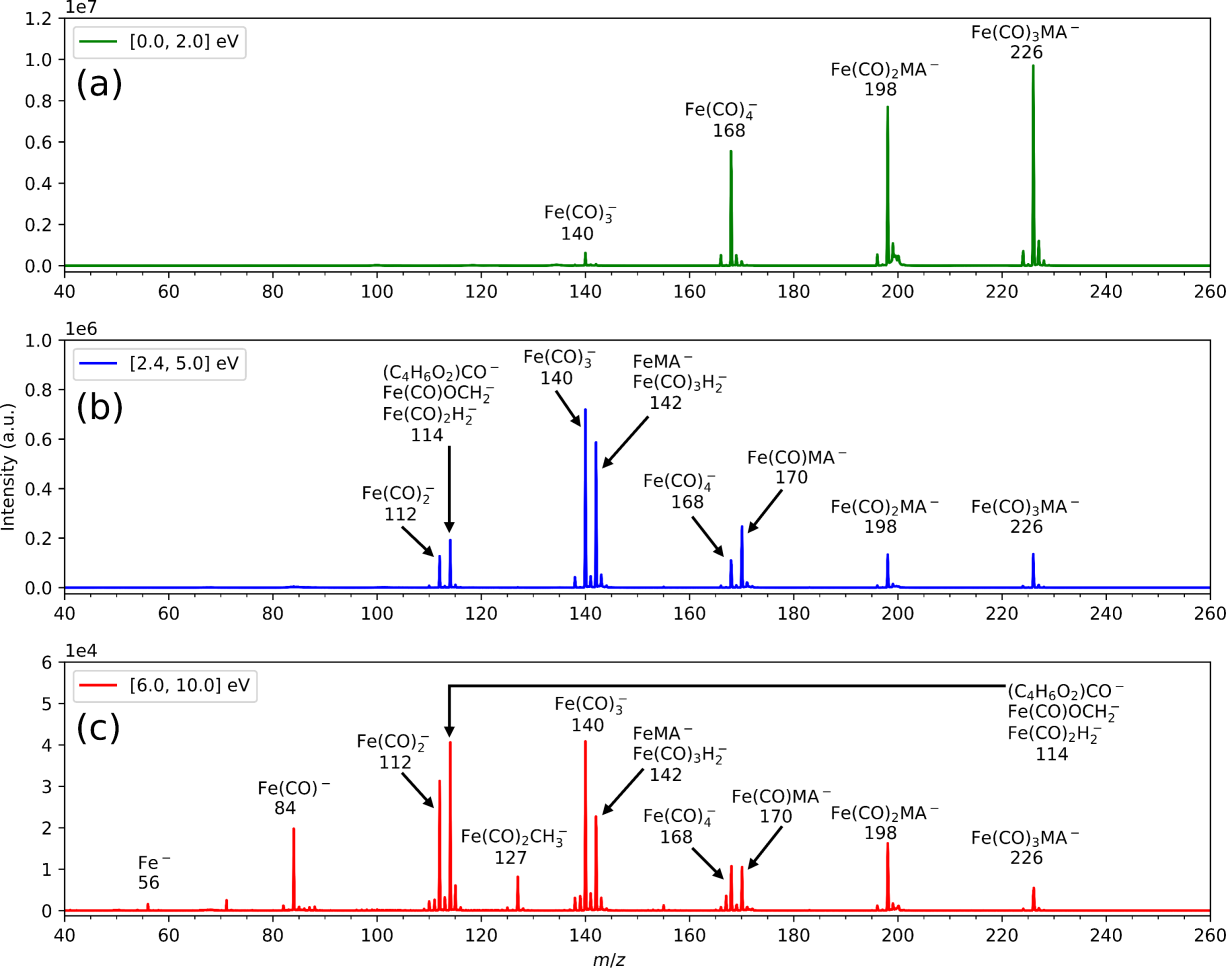
Energy-integrated anionic mass spectra (sum of mass spectra in the given energy range, recorded with steps of 0.1 eV) in three different energy ranges denoted in the individual panels. The spectra were measured using the CLUB experimental setup.

The anion fragmentation pattern changes very much with the incident electron energy. Figure 3 shows the anion mass spectra of Fe(CO)_4_MA measured on the CLUB setup for three different energy ranges. In the low-energy region (Figure 3a shows the sum of the mass spectra between 0 and 2 eV recorded with energy steps of 0.1 eV), the strongest channel is loss of one carbonyl ligand (*m*/*z* = 226), followed in intensity by loss of two carbonyl ligands (*m*/*z* = 198) and loss of the methyl acrylate ligand (*m*/*z* = 168). The channel with the loss of three carbonyl ligands does not appear in this energy range. At higher electron energies (Figure 3b,c), the fragmentation pattern increases, and more complete ligand separation occurs. It is particularly interesting that, with increasing electron energy, the loss of the MA ligand (formation of *m*/*z* = 168, Fe(CO)_4_^−^) is becoming dominant over the loss of one carbonyl ligand (*m*/*z* = 226). There are also fragments visible in which the methyl acrylate ligand is broken, for instance, *m*/*z* = 114 (which can correspond to several different anions) or Fe(CO)_2_CH_3_^−^ (*m*/*z* = 127). In general, the anionic pathways exhibit much less fragmentation than the dissociative ionization ones, that is, Fe^−^ and Fe(CO)^−^ are present in the spectra, however, with rather low abundances.

**Figure 4:**
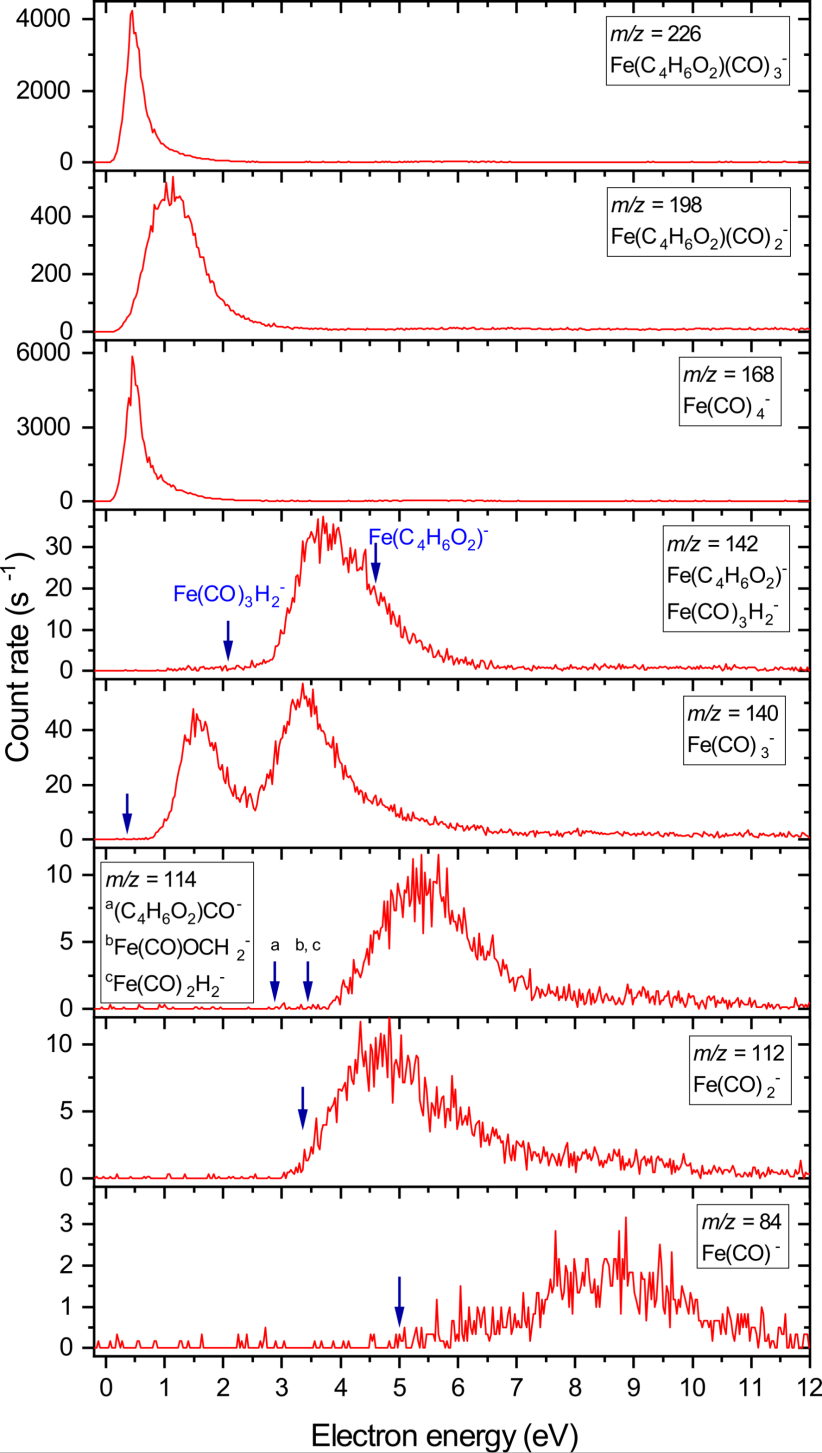
Ion yield curves of DEA of Fe(CO)_4_(C_4_H_6_O_2_) measured on the DEA-QMS setup. The vertical arrows denote the calculated energetic thresholds. For *m*/*z* = 226, 198, and 168, the calculated thresholds are below zero (channels are exothermic); thus, the arrows are not shown.

The strong energy variation is not surprising. DEA is a resonant process; hence, particular fragments are created only at certain energies. Figure 4 shows ion yields (relative cross sections) of selected fragment anions as a function of the electron energy. These spectra were recorded on the TEM-QMS setup, which has a higher energy resolution and more reliable performance at low electron energies than the CLUB setup (as will be demonstrated below). The arrows in Figure 4 denote positions of the calculated thresholds, which are tabulated in Table 2. The two reaction channels that show onsets and peaks close to 0 eV (*m*/*z* = 226, loss of one carbonyl, and *m*/*z* = 168, loss of methyl acrylate) are exothermic by 1.40 and 1.42 eV, respectively. Even though the experimental signal does not peak at 0 eV but somewhat higher, we ascribe this to an instrumental effect, namely, the absence of low-energy electrons in the beam (this was verified by recording the signal of SF_6_^−^ from SF_6_, which peaked at the same energies as the two exothermic channels here). The loss of two carbonyls leading to Fe(CO)_2_MA^−^ is barely exothermic (by 0.02 eV). However, its DEA band looks very different, appearing as a rather broad peak around 1 eV incident energy. The target molecule possesses six unsaturated bonds, which will give rise to low-lying π* shape resonances. These will be closely spaced and overlapping. We thus presume that the 1 eV DEA band originates from this resonance system (in iron pentacarbonyl, the π* resonance system is centered around 1.4 eV [11]). There are additional DEA bands at energies of 3.5, 5.5, and 8.5 eV visible in the other DEA fragments. We ascribe these to core-excited resonances where the electron is temporarily trapped by the excited molecule. The UV–vis spectrum of Fe(CO)_4_MA (shown in Supporting Information File 1) peaks at 267 nm (4.64 eV) and has a visible shoulder around 350 nm (3.54 eV). Even the lowest of these DEA bands can, thus, be assigned to a core-excited resonance.

**Table 2:** Calculated threshold energies (in units of eV) of anionic fragments from the reaction Fe(CO)_4_(η^2^-C_4_H_6_O_2_) + e^−^.

*m*/*z*	Ion	Neutral fragments	*E*_th_

254	Fe(CO)_4_(η^2^-C_4_H_6_O_2_)^−^	—	−1.35^a^
226	Fe(CO)_3_(η^2^-C_4_H_6_O_2_)^−^	CO	−1.40^a^
198	Fe(CO)_2_(η^2^-C_4_H_6_O_2_)^−^	2 × CO	−0.02^a^
170	Fe(CO)(η^2^-C_4_H_6_O_2_)^−^	3 × CO	2.23
168	Fe(CO)_4_^−^	(C_4_H_6_O_2_)	−1.42^a^
142	Fe(η^2^-C_4_H_6_O_2_)^−^	4 × CO	4.35
142	Fe(CO)_3_H_2_^−^	(C_4_H_4_O_2_), CO	2.08
140	Fe(CO)_3_^−^	(C_4_H_6_O_2_), CO	0.36
127	Fe(CO)_2_CH_3_^−^	(C_3_H_3_O_2_), 2 × CO	4.49
114	(C_4_H_6_O_2_)CO^−^	Fe(CO)_3_	2.87
114	Fe(CO)OCH_2_^−^	3 × CO, C_3_H_4_O	3.44
114	Fe(CO)_2_H_2_^−^	2 × CO, (C_4_H_4_O_2_)	3.48
112	Fe(CO)_2_^−^	2 × CO, (C_4_H_6_O_2_)	3.39
86	C_4_H_6_O_2_^−^	Fe(CO)_4_	1.29
84	Fe(CO)^−^	3 × CO, (C_4_H_6_O_2_)	5.00
56	Fe^−^	4 × CO, (C_4_H_6_O_2_)	7.68

^a^Exothermic reaction.

An interesting finding is the nature of *m*/*z* = 142, which could in principle correspond to the removal of all four carbonyl ligands and the formation of FeMA^−^. However, the calculated threshold for this process is 4.35 eV. The DEA signal is already appearing between 2 and 3 eV electron energy, and it peaks at 3.7 eV. The alternative fragment with this mass is Fe(CO)_3_H_2_^−^, in which two H atoms are transferred from the MA ligand to the iron atom. This channel has a calculated threshold of 2.08 eV; the recorded signal can, thus, indeed originate from Fe(CO)_3_H_2_^−^. Unfortunately, such a distinction is not possible for *m*/*z* = 114 since the calculated thresholds for all three possible structures are below the onset of the experimental signal.

Again, a comparison with DEA of gas-phase iron pentacarbonyl [11] might be instructive. There, the only exothermic channel is the dissociation of one carbonyl ligand, leading to a high Fe(CO)_4_^−^ yield at low electron energies. In the present case, the loss of either a carbonyl or the methyl acrylate ligand is exothermic, and this is manifested in the near-zero-electronvolts DEA peaks. High-resolution DEA studies, together with a use of effective range theory with complex boundary conditions, have shown that in Fe(CO)_5_ a crucial factor influencing the low-energy DEA is the long-range electron–molecule interaction [11]. In nonpolar Fe(CO)_5_, this is mediated by a high polarizability. The calculated isotropic polarizability of Fe(CO)_4_MA (144 a.u.) is lower than that of Fe(CO)_5_ (189 a.u.); however, it is a polar molecule with a calculated total dipole moment of 1.72 Debye. We thus presume that, also in the present case, the high DEA cross section close to 0 eV is mediated by long-range electron–precursor interactions to a large extent.

For higher electron energies, the resonance structures in Fe(CO)_5_ and Fe(CO)_4_MA are very similar. There is the already mentioned π* shape resonance around 1 and 1.4 eV, respectively, which leads to the dissociation of two ligands (in the present case either two CO ligands or one CO and one MA ligand). Also, there are several core-excited resonances that lead to more complete stripping. In both cases, the bare Fe^−^ anion is observed at electron energies around 8 eV, albeit with very low intensities.

**Figure 5:**
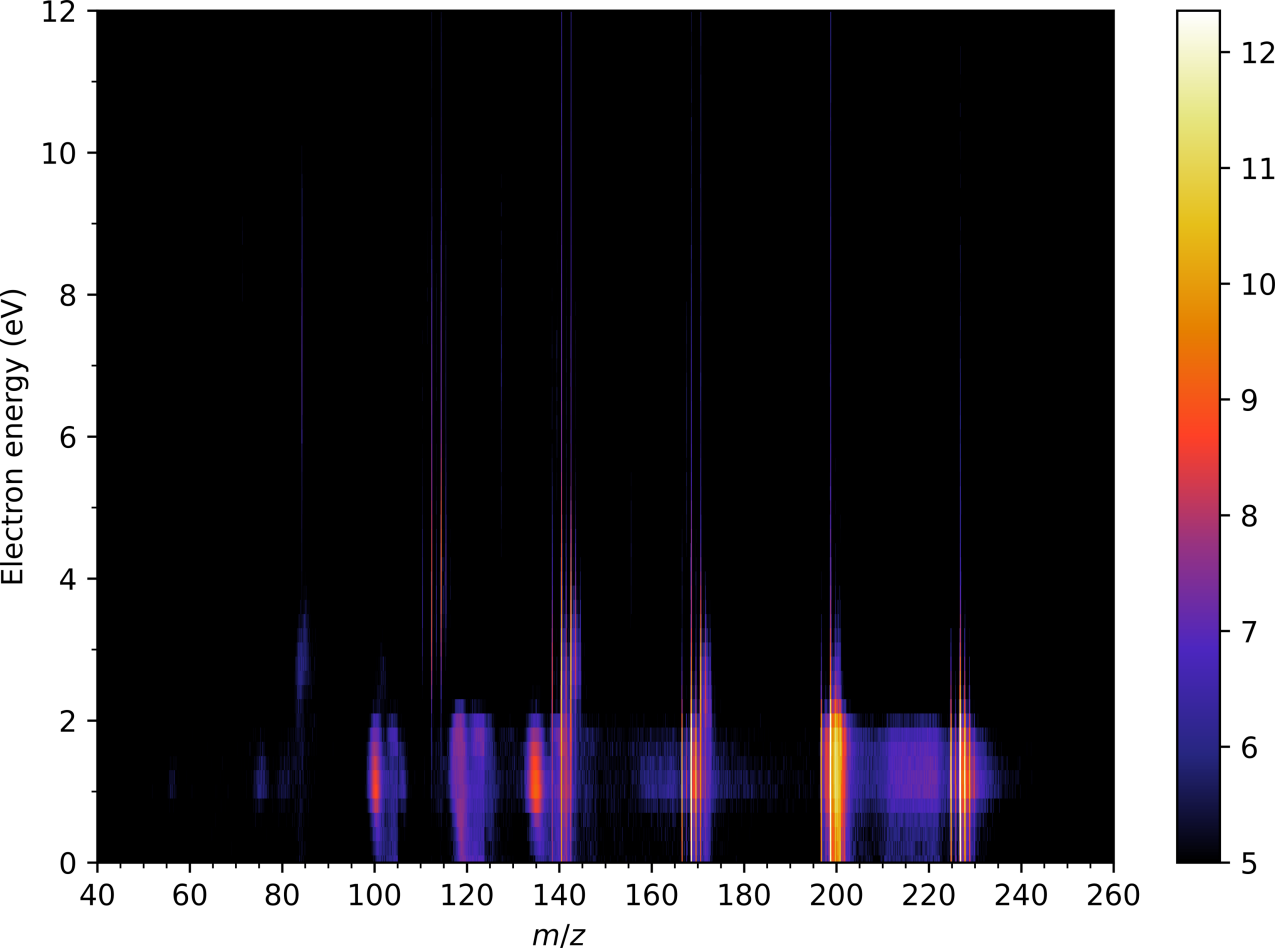
2D plot of the dependence of negative ion mass spectra on the incident electron energy measured using the CLUB setup. The logarithm of the ion intensity is color-coded as shown by the color bar on the right.

Finally, we would like to discuss the anionic 2D map measured on the CLUB setup (anion mass spectra as a function of electron energy). The map is shown in Figure 5. In general, there are two types of recognizable features. The thin vertical traces correspond to a “well-behaved” signal, and the intensity profiles along them agree very well with the ion yields from Figure 4. However, in addition to these, there is a group of features located at electron energies below 2 eV, which span over a range of *m*/*z* values. Since these signals were not confirmed on the TEM-QMS setup, we consider them as unphysical and as setup-specific experimental artifacts. We decided to show them here basically as a demonstration of how easy it can be to create spurious low-energy peaks in DEA experiments. We can only speculate about the origin of these low-energy signals on CLUB. There is a physical process that can contribute to their appearance, namely, metastability of the transient anions on the microsecond time scale. CLUB is equipped with a reflectron time-of-flight mass analyzer consisting of an extraction region, the first field-free region, the reflection ion optic, the second field-free region, and a detector. If a parent anion is formed and it decays at various later stages of its flight through the setup, it can create signals either between the parent and the fragment ions [38–39] (if the decay happens in the first field-free region) or lead to the broadening of the ion mass peaks (if the decay happens in the extraction region). Non-physical causes of these spurious signals can be related either to the collision-induced dissociation of the weakly bound anions in the mass analyzer or to a significant broadening of the electron beam at low electron energies (such that the ions are produced in such a large volume that the RTOF is not able to time-focus them). It is also important to note that these signals are rather low and are visually amplified by the logarithmic color scale used in Figure 5 (they are not visible in the linear scale of Figure 3a, which was also recorded on the CLUB setup at low electron energies).

## Conclusion

We report on electron-induced fragmentation of Fe(CO)_4_MA with a view of its possible use as a nanofabrication precursor. Dissociative ionization of this molecule leads to extensive fragmentation with bare Fe^+^ and Fe(CO)^+^ being the dominant fragments. However, the dominance of these stripped ions is not as pronounced as in the case of iron pentacarbonyl. For dissociative electron attachment, the fragmentation pattern changes strongly with the electron energy. At very low energies, the precursor efficiently loses either one carbonyl or one MA ligand, and the fragmentation is more complete at increased electron energies.

The present data can be used to interpret the results of an electron-induced deposition using Fe(CO)_4_MA [8]. There, it has been concluded that electron irradiation efficiently separates the neutral MA ligand from the precursor. This suggests that dissociative ionization plays only a limited role in the deposition process since the Fe(CO)_4_^+^ fragment has a very low abundance in the mass spectrum. In contrast, the MA ligand is very efficiently cleaved in the dissociative electron attachment at very low electron energies. This is in accordance with the observations in [8] that deposits of similar purity were obtained from Fe(CO)_5_ and Fe(CO)_4_MA despite the higher number of C and O atoms in MA, and that deposits of higher purity were obtained under conditions yielding a large number of secondary electrons, which could induce MA loss by dissociative electron attachment.

## Supporting Information

Supporting Information contains details of the DFT calculation, ion yield curves for dissociative ionization, and FTIR, NMR and UV–vis spectra of the sample.

File 1 Additional experimental data.

## Data Availability

The data that supports the findings of this study is available from the corresponding author upon reasonable request.
