# Supplementary material for: Electron-induced ligand loss from iron tetracarbonyl methyl acrylate
Source: Beilstein J Nanotechnol. 2024 Jul 3;15:797–807. doi: 10.3762/bjnano.15.66 (PMC11228821; doi:10.3762/bjnano.15.66)
Supplement: File 1 — Additional experimental data. [file Beilstein_J_Nanotechnol-15-797-s001.pdf]

BEILSTEIN JOURNAL OF NANOTECHNOLOGY

Supporting Information

for

Electron-induced ligand loss from iron tetracarbonyl methyl acrylate

Hlib Lyshchuk, Atul Chaudhary, Thomas F. M. Luxford, Miloš Ranković, Jaroslav Kočišek, Juraj Fedor, Lisa McElwee-White and Pamir Nag

Beilstein J. Nanotechnol. **2024,** *15,* 797–807. doi:10.3762/bjnano.15.66

Additional experimental data

License and Terms: This is a supporting information file under the terms of the Creative Commons Attribution License (https://creativecommons.org/licenses/by/4.0). Please note that the reuse, redistribution and reproduction in particular requires that the author(s) and source are credited and that individual graphics may be subject to special legal provisions.
The license is subject to the *Beilstein Journal of Nanotechnology* terms and conditions: (https://www.beilstein-journals.org/bjnano/terms)

DFT calculations

We performed DFT-based structure optimization calculations using Gaussian 16 software [1]. Fragments with an even number of electrons were assumed to be in singlet spin states, while fragments with an odd number of electrons were assumed to be in doublet spin states. All calculations were conducted using the commonly employed hybrid functional B3LYP [2] with a 6-31++G(d,p) [3,4] basis set and included the GD3 empirical dispersion correction [5]. The different anions and cations formation channels and the corresponding appearance energies are shown in Table S1 and Table S2, respectively. Structures of the fragments that are not obvious from the chemical formula alone are also shown in the tables. The coordinates of the optimized structure of neutral $Fe(CO)_4(\eta^2\text{-}C_4H_6O_2)$ are presented in Table S3, and visualizations of the HOMO and HOMO-1 orbitals are shown in Figure S1.

Table S1: B3LYP/6-31++G(d,p) threshold energies for individual fragmentation channels of the cations. The inset pictures show molecules or fragments whose structure is not obvious from the chemical formula.

Products			*m/z*	E_{th}
$Fe(CO)_4(\eta^2\text{-}C_4H_6O_2)^+$ 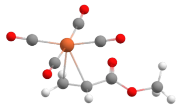			254	7.43
$Fe(CO)_3(\kappa^1\text{-}C_4H_6O_2)^+$ 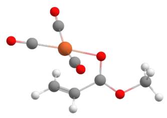	+	CO	226	7.96
$Fe(CO)_3(\kappa^1\text{-}C_4H_6O_2)^+$ 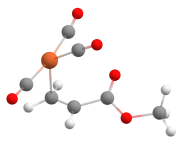	+	CO	226	8.75

Table S1 (Continued)

**Products**		***m/z***	$\boldsymbol{E_{th}}$
$Fe(CO)_2(\eta^4\text{-}C_4H_6O_2)^+$ 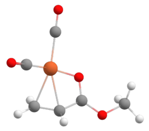	+ 2 x CO	198	8.62
$Fe(CO)_2(\kappa^1\text{-}C_4H_6O_2)^+$ 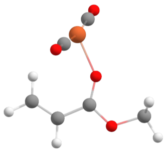	+ 2 x CO	198	9.33
$Fe(CO)(\eta^4\text{-}C_4H_6O_2)^+$ 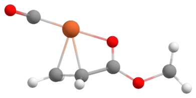	+ 3 x CO	170	10.06
$Fe(CO)(\eta^2\text{-}C_4H_6O_2)^+$ 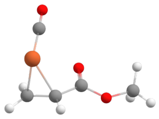	+ 3 x CO	170	11.31
$Fe(CO)_4^+$	+ $C_4H_6O_2$ 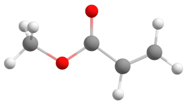	168	8.91
$Fe(\eta^4\text{-}C_4H_6O_2)^+$ 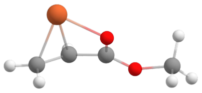	+ 4 x CO	142	11.62
$Fe(CO_3)H_2^+$ 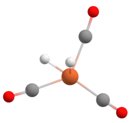	+ CO + $C_4H_4O_2$ 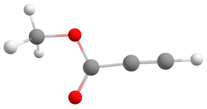	142	13.01
$Fe(CO)_3^+$	+ $C_4H_6O_2$ + CO	140	10.04

Table S1 (Continued)

**Products**		***m/z***	$\boldsymbol{E_{th}}$
$Fe(CO)_2CH_3^+$	+ $C_3H_3O_2$ + 2 x CO	127	13.33
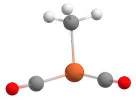	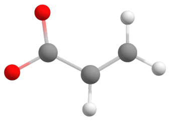		
$(C_4H_6O_2)CO^+$	+ $Fe(CO)_3$	114	11.21
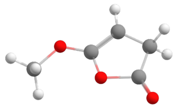			
$Fe(CO)OCH_2^+$	+ C_3H_4O + 3 x CO	114	14.90
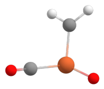	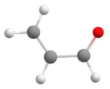		
$Fe(CO)_2H_2^+$	+ 2 x CO + $C_4H_4O_2$	114	15.22
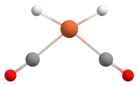			
$Fe(CO)_2^+$	+ $C_4H_6O_2$ + 2 x CO	112	12.31
$(C_4H_6O_2)^+$	+ $C_4H_6O_2$ + 2 x CO	86	11.07
$Fe(CO)^+$	+ $C_4H_6O_2$ + 3 x CO	84	14.20
Fe^+	+ $C_4H_6O_2$ + 4 x CO	56	15.65

Table S2: B3LYP/6-31++G(d,p) threshold energies for individual fragmentation channels of the anions. The inset pictures show molecules or fragments whose structure is not obvious from the chemical formula.

**Products**	***m/z***	$\boldsymbol{E_{th}}$
$Fe(CO)_4(\eta^2\text{-}C_4H_6O_2)^-$	254	−1.35*
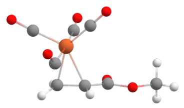		

Table S2 (Continued)

**Products**		***m/z***	$\boldsymbol{E_{th}}$
$Fe(CO)_3(\eta^2\text{-}C_4H_6O_2)^-$	+ CO	226	−1.40
$Fe(CO)_2(\eta^2\text{-}C_4H_6O_2)^-$	+ 2 x CO	198	−0.02
$Fe(CO)(\eta^2\text{-}C_4H_6O_2)^-$	+ 3 x CO	170	2.23
$Fe(CO)_4^-$	+ $C_4H_6O_2$	168	−1.42
$Fe(\eta^2\text{-}C_4H_6O_2)^-$	+ 4 x CO	142	4.35
$Fe(CO_3)H_2^-$ 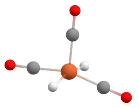	+ CO + $C_4H_4O_2$ 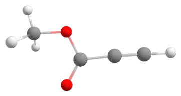	142	2.08
$Fe(CO)_3^-$	+ $C_4H_6O_2$ + CO 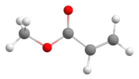	140	0.36
$Fe(CO)_2CH_3^-$ 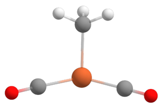	+ $C_3H_3O_2$ + 2 x CO 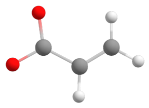	127	4.49
$(C_4H_6O_2)CO^-$ 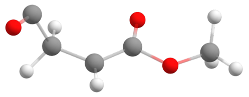	+ $Fe(CO)_3$	114	2.87
$Fe(CO)OCH_2^-$ 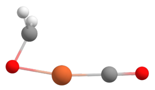	+ C_3H_4O+ 3 x CO 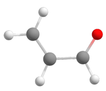	114	3.44
$Fe(CO)OCH_2^-$ 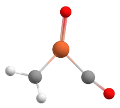	+ C_3H_4O+ 3 x CO 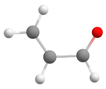	114	4.20

Table S2 (Continued)

**Products**		***m/z***	***E_{th}***
$Fe(CO)_2H_2^-$ 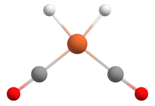	+ 2 x CO + $C_4H_4O_2$	114	3.48
$Fe(CO)_2^-$	+ $C_4H_6O_2$ + 2 x CO	112	3.39
$C_4H_6O_2^-$	+ $C_4H_6O_2$ + 2 x CO	86	1.29
$Fe(CO)^-$	+ $C_4H_6O_2$ + 3 x CO	84	5.00
Fe^-	+ $C_4H_6O_2$ + 4 x CO	56	7.68

* The electron affinity value for $Fe(CO)_4(\eta^2\text{-}C_4H_6O_2)$.

Table S3: XYZ Coordinates of the optimized structure of $Fe(CO)_4(\eta^2\text{-}C_4H_6O_2)$.

Atom	X (Å)	Y (Å)	Z (Å)
O	-2.881187	0.910581	-0.077459
O	-2.357450	-1.175561	-0.783833
C	-4.128350	0.395841	0.429466
C	-2.066167	0.002910	-0.661827
C	-0.807439	0.637590	-1.123721
C	0.102051	-0.063763	-1.947829
H	-4.640318	1.253743	0.864332
H	-3.942974	-0.369939	1.187387
H	-4.718889	-0.039505	-0.380634
H	-0.852127	1.721935	-1.154158
H	0.733522	0.484533	-2.639209
H	-0.140561	-1.077799	-2.250118
Fe	0.876977	0.004260	0.026817
C	0.429693	0.481412	1.717303
C	2.521215	-0.725372	-0.114611
C	1.599053	1.647123	-0.301881
C	0.221348	-1.700501	0.305875
O	0.137533	0.778933	2.790714
O	2.052368	2.680092	-0.516786
O	3.576254	-1.180227	-0.205949
O	-0.086081	-2.780716	0.525627

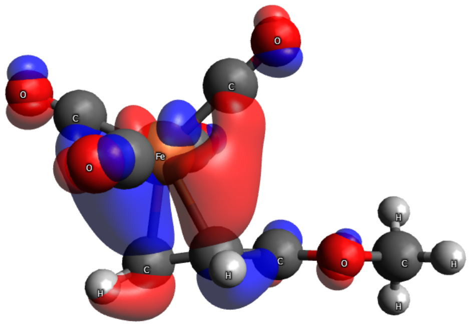

(a) HOMO

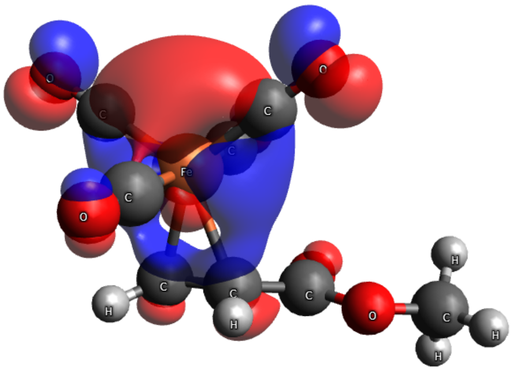

(b) HOMO-1

Figure S1: Visualization of (a) HOMO and (b) HOMO-1 orbitals of $Fe(CO)_4(\eta^2{-}C_4H_6O_2)$.

Ion yield curves in dissociative ionization

The dependence of the intensity of a given *m*/*z* peak on the electron energy is shown in Figure S2 for five cationic fragments.

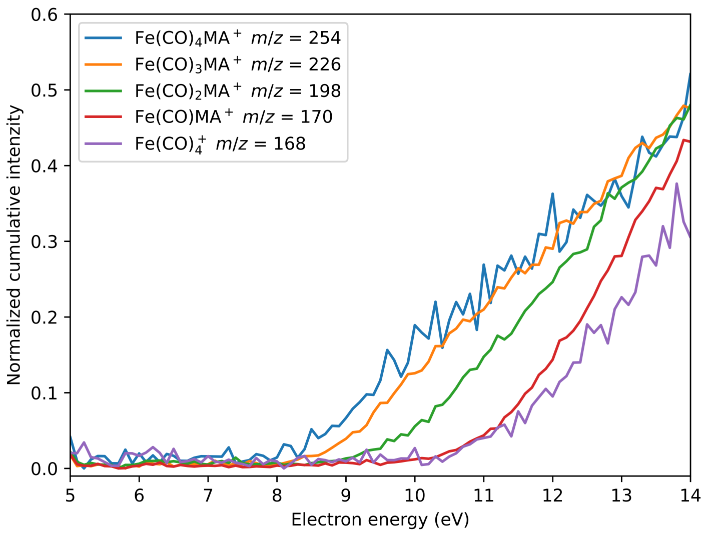

Figure S2: Ion yield curves for five cationic fragments of $Fe(CO)_4(C_4H_6O_2)$.

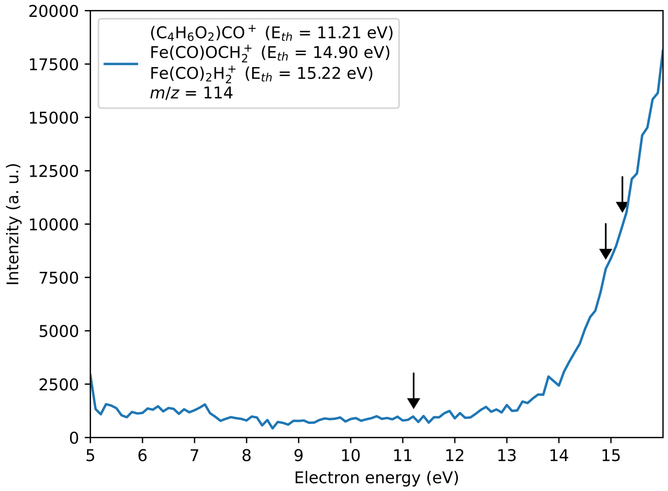

Figure S3: Ion yield curve for *m/z* = 114. The probable chemical structure can be $(C_4H_6O_2)CO^+$, $Fe(CO)OCH_2^+$, or $Fe(CO)_2H_2^+$.

FTIR spectrum

The Fourier-transform infrared spectrum of iron tetracarbonyl methyl acrylate, $Fe(CO)_4MA$, measured in hexane solution is shown in Figure S4. The spectrum was obtained on a PerkinElmer Spectrum ONE FTIR spectrometer using a solution cell equipped with NaCl windows and a path length of 1.0 mm.

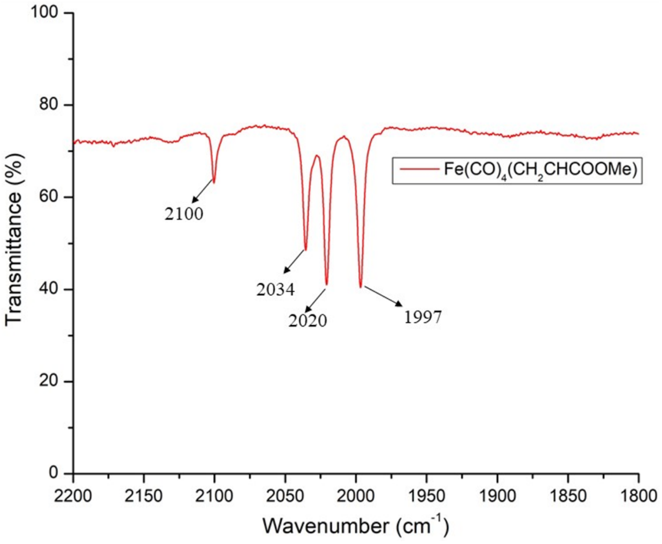

Figure S4: FTIR spectrum of $Fe(CO)_4MA$ (MA = methyl acrylate, $H_2C{=}CH\text{-}COOCH_3$) obtained in hexane solution: ν_{CO} (cm^{-1}): 2100, 2034, 2020, 1997.

NMR spectrum

The ^{1}H NMR spectrum of iron tetracarbonyl methyl acrylate, $Fe(CO)_4MA$, is shown in Figure S5. The spectrum was obtained on a Bruker 400 MHz spectrometer at room temperature.

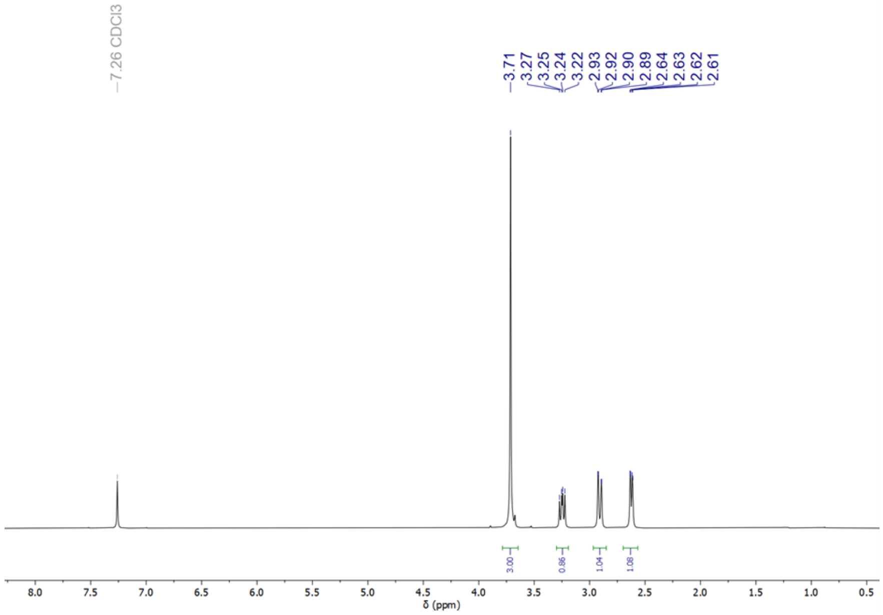

Figure S5: ^{1}H NMR (400 MHz, 25°C, $CDCl_3$) spectrum of $Fe(CO)_4MA$ (MA = methyl acrylate, $H_2C{=}CH\text{-}COOCH_3$).

UV–vis spectrum

The UV–vis absorption spectrum of iron tetracarbonyl methyl acrylate was measured using a Shimazdu UV-1650PC UV–vis absorption spectrometer. The experiment was performed in a cuvette of 10 mm path length. The sample was dissolved in hexane. The UV–vis spectrum is shown in Figure S6. The molar extinction coefficient was 5669 $M^{-1}cm^{-1}$. The absorption maximum is at around 267 nm, that is, 4.64 eV. Also, a shoulder around 350 nm (3.54 eV) can be observed on top of the broad peak.

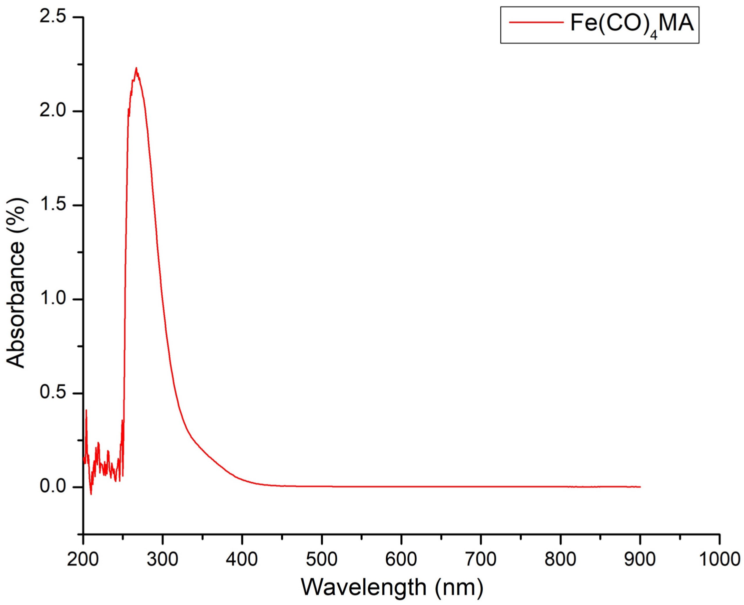

Figure S6: UV–vis absorption spectrum of iron tetracarbonyl methyl acrylate.

References

1. *Gaussian 16*, ES64L-G16RevC.01; Gaussian, Inc.: Wallingford, CT, USA, 2016.

2. Becke, A. D. *J. Chem. Phys.* **1993**, *98*, 1372–1377. doi:10.1063/1.464304.

3. Petersson, G. A.; Bennett, A.; Tensfeldt, T. G.; Al-Laham, M. A.; Shirley, W. A.; Mantzaris, J. *J. Chem. Phys.* **1988**, *89*, 2193–2218. doi:10.1063/1.455064.

4. Petersson, G. A.; Al-Laham, M. A. *J. Chem. Phys.* **1991**, *94*, 6081–6090. doi:10.1063/1.460447.

5. Grimme, S.; Antony, J.; Ehrlich, S.; Krieg, H. *J. Chem. Phys.* **2010**, *132*, 154104. doi:10.1063/1.3382344.
